# Molecular Mechanism of Matrine from *Sophora alopecuroides* in the Reversing Effect of Multi-Anticancer Drug Resistance in K562/ADR Cells

**DOI:** 10.1155/2019/1269532

**Published:** 2019-11-22

**Authors:** Zhi Chen, Nobuhiro Nishimura, Takayuki Okamoto, Koichiro Wada, Kohji Naora

**Affiliations:** ^1^Department of Pharmacy, Shimane University Hospital, Izumo, Shimane, Japan; ^2^Department of Pharmacology, Faculty of Medicine, Shimane University, Izumo, Shimane, Japan; ^3^School of Pharmaceutical Sciences, International University of Health and Welfare, Okawa, Fukuoka, Japan

## Abstract

Multidrug resistance is the main obstacle to current chemotherapies. In this study, we evaluated the reversing effect of matrine, the principal alkaloid derived from *Sophora alopecuroides*, on chemoresistant leukemia K562/ADR cells. Matrine in a range of the nontoxic concentration was employed in the whole study. IC_50_s of cancer medicines were tested using WST-8 assay. Drug export and apoptotic rates were examined using flow cytometry. The mRNA and protein expressions were quantified by quantitative real-time PCR and western blotting, respectively. Our data indicated that matrine had potent reversal properties augmenting cytotoxicity of cancer medicines on K562/ADR cells as well as apoptotic rates induced by doxorubicin. Moreover, matrine inhibited drug-exporting activity and expression of ATP-binding cassette subfamily B member 1 (ABCB1) on both mRNA and protein levels. That might result from inhibited NF-kappa B activation, which also led to restored intrinsic apoptosis. These findings suggest that matrine in the nontoxic concentration can suppress ABCB1 drug transport and facilitate the intrinsic apoptosis pathway through the inhibiting effect on NF-kappa B and has the potential to become an efficient sensitizer for anticancer drug resistance.

## 1. Introduction

**Figure 1:**
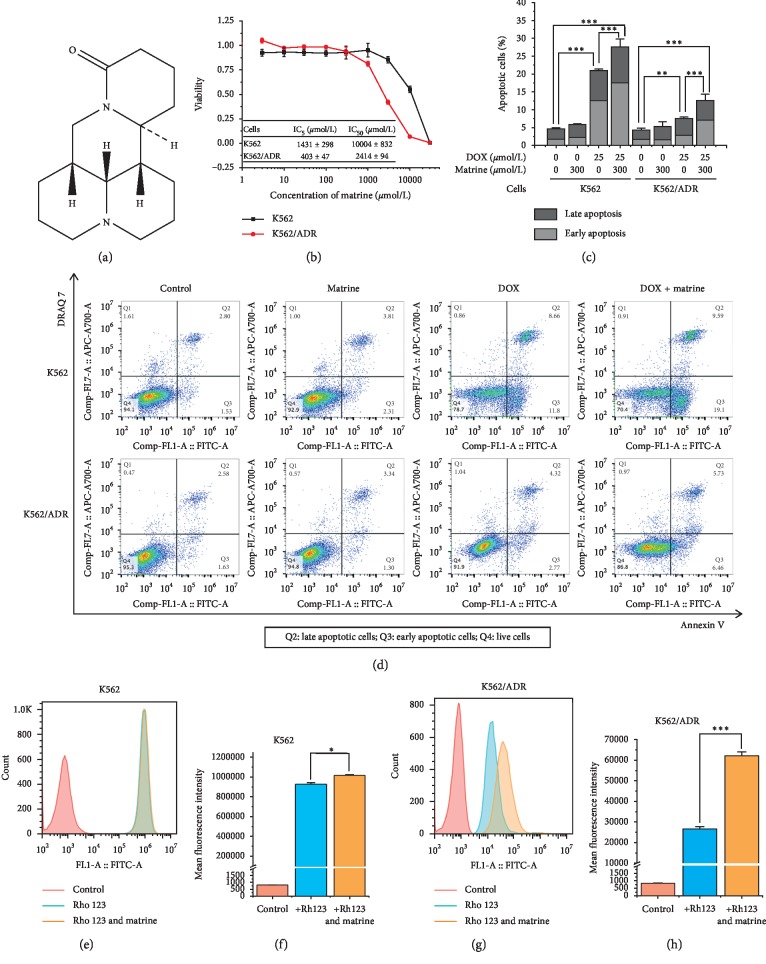
The effect of matrine in the nontoxic concentration on apoptosis and rhodamine 123 accumulation in K562 and K562/ADR cells. (a) Chemical structure of matrine. (b) Evaluation of the nontoxic concentration of matrine carried out by WST-8 assay. (c) and (d) Enhancement of DOX-induced apoptosis by matrine, measured by Annexin V-FITC and DRAQ 7 staining with flow cytometry. (e), (f), (g), and (h) Change in intracellular rhodamine 123 (Rho 123) accumulation by matrine, examined by flow cytometry. All the data were expressed as mean and SD of three independent experiments ((b), (c), (f), and (h); ^*∗*^*P* < 0.05, ^*∗∗*^*P* < 0.01, ^*∗∗∗*^*P* < 0.001; ANOVA or *t*-test).

Matrine is the main alkaloid derived from herbal medicine *Sophora alopecuroides* (Figure 1(a)). Its antitumor effect has been widely concerned in recent years [1]. Many researchers [2, 3] have revealed that matrine has a cytotoxic effect on cancer cells due to inhibiting the proliferation of the cells and inducing apoptosis. Now an anticancer injection containing matrine named “Compound Kushen Injection” [4] has been applied clinically in China. Clinical assessment of efficacy and safety on breast cancer was also implemented [5]. Recently, some research studies indicated that matrine might have more valuable properties that sensitize resistant cancer cells to chemotherapeutic agents through suppressing drug exporter ABCB1 and inhibitor of *κ*B kinase *β*, which is an NF-kappa B activator [6, 7]. However, there is still no systematic research illustrating the mechanism for suppressing the effect of matrine on drug resistance.

Multidrug resistance is a notorious mechanism that induces cancer cells to develop resistance against chemotherapies [8]. Changes in drug export and the apoptosis pathway constitute the primary molecular mechanisms being responsible for the resistance of tumors to anticancer agents [9]. Drug transport associated with multidrug resistance is mainly the export of anticancer drugs from cells inside by adenosine triphosphate-binding cassette (ABC) transporters [10]. Especially, activation of ABCB1 is a common mechanism for cellular resistance to doxorubicin (DOX), paclitaxel (PTX), and vinblastine [11, 12]. It is noticeable that relevant research had found that silencing NF-kappa B can attenuate activation of ABCB1 [13]. Deregulated apoptosis may be responsible for multidrug resistance in cancer therapy [14]. Intrinsic apoptosis is involved in the cleavage of caspase-3 and caspase-9 [15]. B-cell lymphoma 2 (Bcl-2) protein family is playing critical roles in regulating intrinsic apoptosis [16]. Therefore, NF-kappa B, as an upstream protein of Bcl-2 proteins, can inhibit the intrinsic apoptotic pathway [17, 18]. Collectively, activation of NF-kappa B can probably simultaneously induce drug export and hamper apoptosis, leading to drug resistance.

In this study, we aimed at clarifying if the nontoxic concentration of matrine could enhance the anticancer drugs in multidrug-resistant cells. Moreover, we also attempt to figure out the mechanism by which matrine restores multidrug resistance. Finally, the effects of matrine on the expression and function of drug-exporting transporters and apoptosis-relating proteins were examined.

## 2. Materials and Methods

### 2.1. Cell Culture and Chemicals

The human leukemia cell line K562 and its multidrug-resistant subtype K562/ADR were purchased from Riken Cell Bank (Saitama, Japan). The cells were maintained in RPMI-1640 medium (FUJIFILM Wako Pure Chemical Corporation, Osaka, Japan) supplemented with 10% fetal bovine serum (FBS; Biosera, Nuaillé, France) and 1% penicillin-streptomycin solution (FUJIFILM Wako Pure Chemical Corporation) at 37°C under humidified air with 5% CO_2_. For sustaining the drug-resistant property, 0.7 *μ*mol/L DOX (Sigma-Aldrich, Delhi, India) was supplemented in the medium for K562/ADR until at least 14 days before each experiment. Matrine was obtained from Indofine Chemical Company, Inc. (Hillsborough, New Jersey, USA).

### 2.2. Cell Viability and Reversal Effect Assay

K562 and K562/ADR cells were placed at an initial density of 5000 cells per well into 96-well plates. After 24 h preincubation, cells were implemented with DOX and matrine treatment at indicated concentrations for 48 h. The viable cells were counted using Cell Counting Kit-8 staining according to the manufacturer's instructions (Dojindo Molecular Technologies, Kumamoto, Japan). Absorbance was read by Multimode Detector DTX 880 (Beckman Coulter, Tokyo, Japan). The concentrations required to inhibit growth by 50% (IC_50_) were calculated from viability curves using GraphPad Prism 8 (San Diego, California, USA). Reversal fold [19, 20] values were calculated using the following formula: reversal fold = IC_50_ of DOX alone/IC_50_ of DOX in the presence of matrine, to assess the effect of matrine on multidrug resistance. All experiments were performed three times.

### 2.3. RNA Extraction and Real-Time Quantitative RT-PCR

K562 and K562/ADR cells were seeded into six-well flat-bottom plates and cultured overnight in RPMI-1640 medium supplemented with 10% FBS, followed by being treated with or without DOX or matrine at the indicated concentration for 48 h in the dark at 37°C with 5% CO_2_. Cells were harvested and washed by ice-cold PBS twice (FUJIFILM Wako Pure Chemical Corporation). Total RNA was extracted with the RNeasy Mini Kit (QIAGEN, Tokyo, Japan). The primers for ABCB1, ABCC1, ABCG2, phosphatase and tensin homolog (PTEN), p53, mouse double minute 2 homolog (MDM2), Akt, and GAPDH were provided by Eurofins Genomics (Tokyo, Japan) and are shown in [Supplementary-material supplementary-material-1]. The first-strand cDNA was synthesized with the ReverTra Ace qPCR RT Master Mix with gDNA Remover (TOYOBO CO., LTD., Osaka, Japan). Quantitative RT-PCR was performed with KOD SYBR qPCR Mix (TOYOBO CO., LTD.) and measured in the Thermo Cycler Dice™ Real-Time System TP900 (TAKARA Bio Inc., Shiga, Japan). GAPDH was used as a housekeeping control. The relative amount of target mRNA was determined using the 2^−ΔΔ*Ct*^ assay [21]. All experiments were triplicated.

### 2.4. Western Blotting

Cell culture was the same as previously described. Whole-cell lysates were prepared for western blotting as we previously performed [22]. The samples (2 *μ*g for ABCB1 measurement, 30 *μ*g for others) were equally subjected to 4–15% Mini-PROTEAN TGX Precast Protein Gels (Bio-Rad, Hercules, California, USA) and transferred to Immun-Blot PVDF Membrane (Bio-Rad). After blocking with Blocking One buffer (Nacalai Tesque, Inc., Kyoto, Japan) for 2 h, the membranes were incubated with the indicated antibodies for overnight at 4°C, followed by incubation with horseradish peroxidase-conjugated secondary antibody for 2 h at room temperature. The bands were further dealt with Chemi-Lumi One L solution (NACALAI) and analyzed. The primary antibodies for caspase-8 were obtained from Medical & Biological Laboratories CO., LTD. (Nagoya, Japan) and those for caspase-3, caspase-9, survivin, NF-kappa B p65, p-NF-kappa B p65, Bcl-xL, and Bcl-2-associated X protein (Bax) were from Cell Signaling Technology, Inc. (Danvers, Massachusetts, USA). Primary antibodies for ABCB1 and GAPDH and secondary antibodies goat anti-rabbit IgG H&L (HRP) and rabbit anti-mouse IgG H&L (HRP) were purchased from Abcam (Cambridge, Massachusetts, USA). The density of bands was measured using a LAS4000 fluorescence image analysis system (FUJIFILM, Tokyo, Japan). All experiments were performed three times.

### 2.5. Apoptosis Assay

Cell apoptosis was evaluated with flow cytometry. K562 and K562/ADR cells were seeded into six-well flat-bottom plates and cultured overnight in RPMI-1640 medium supplemented with 10% FBS, followed by being treated with or without DOX or matrine at the indicated concentration for 16 h in the dark at 37°C with 5% CO_2_. Cells were harvested and washed twice with PBS, stained with Annexin V-FITC (Abcam) and DRAQ7 (BioLegend, San Diego, California, USA) in the binding buffer (Abcam), and detected by CytoFLEX flow cytometer (Beckman Coulter, Tokyo, Japan) after 5-minute incubation at room temperature in the dark. Fluorescence was determined with *λ*ex 488 nm through FITC channel (525/40 nm) for Annexin V-FITC and λex 638 nm through APC-700 channel (712/25 nm) for DRAQ7. The early apoptotic cells (Annexin V positive only) and late apoptotic cells (Annexin V and DRAQ7 positive) were quantified. All experiments were performed three times.

Annexin V-FITC/propidium iodide (PI) staining is a typical assay for dividing the live, early apoptotic, and late apoptotic cells using flow cytometry [11, 23]. However, in our study, a high concentration of DOX is required to induce apoptosis in our K562/ADR cells due to its strong resistance against DOX caused by constantly cocultured with low-concentration DOX. The concentration of DOX (*λ*ex = 480–500 nm and *λ*em = 520–720 nm) [24] used when measuring apoptosis could cause a severe overlapping signal of PI (*λ*ex = 493 nm and *λ*em = 636 nm), which seriously influenced the definition of that PI positive or negative (data not shown). DRAQ 7 (*λ*ex = 633 nm and *λ*em = 695 nm) is a membrane-impermeable dye that only stains the nuclei of dead or permeabilized cells without the interference of DOX. Therefore, we replaced PI with DRAQ 7 to label the late apoptotic cells.

### 2.6. Intracellular Rhodamine 123 Accumulation

The drug export function of ABCB1 was evaluated by measuring the intracellular accumulation of rhodamine 123. Cell culture was the same as previously described. 10 *μ*mol/L rhodamine 123 (Thermo Fisher Scientific, Tokyo, Japan) was added to the wells and incubated for another 1 h. The cells were harvested and washed twice with ice-cold PBS (FUJIFILM Wako Pure Chemical Corporation). The mean fluorescence intensity (MFI) associated with rhodamine 123 was then determined with *λ*ex 488 nm and *λ*em 525 nm using CytoFLEX flow cytometer (Beckman Coulter, Tokyo, Japan). All experiments were performed three times.

### 2.7. Statistics

All the data were statistically analyzed using SPSS 20.0 (Chicago, Illinois, USA), presented as mean and standard deviation, and compared using independent *t*-test (2-tailed) or one-way analysis of variance. *P* < 0.05 was considered statistically significant.

## 3. Results

### 3.1. Determination of the Noncytotoxic Concentration of Matrine

To test the nontoxic concentration range in K562 cells and its chemoresistant subtype K562/ADR cells, firstly, we examined the cytotoxicity of matrine for 48 h by WST-8 assay. The suppressive effect of matrine on the growth rate of K562/ADR cells was stronger than that on the parental cells. IC_50_ of matrine on K562 cells was 3.14-fold higher than on K562/ADR cells. IC_5_s of matrine on the two kinds of cells were regarded as the maximum value of the nontoxic concentration (Figure 1(b)). Therefore, we set 300 *μ*mol/L as the maximum concentration of matrine, which ensures that over 95% of the two kinds of cells would be viable at the same time, in all the following experiments.

### 3.2. Reversal Effect of Matrine on Multidrug Resistance

Based on the nontoxic concentration range of matrine, cytotoxicity of DOX or PTX in the combination of matrine for 48 h was tested. The reversal fold value represents how much resistance in K562/ADR cells was reversed. As shown in Table 1, in contrast to K562 cells, K562/ADR cells had exhibited much stronger resistance against DOX. In the combination of 200 and 300 *μ*mol/L matrine, reversal fold values in K562/ADR cells were 2.30 and 2.88, respectively, indicating more than doubled sensitization to DOX. In contrast, no effect of matrine on the IC_50_s of DOX in the parental cells was observed. This trend was also found in the IC_50_s of PTX (Table 1).

### 3.3. Enhancement of DOX-Induced Apoptosis by Matrine

Effects of matrine in the combination of DOX on the cell apoptosis in the multidrug-resistant cells are shown in Figures 1(c) and 1(d). The apoptotic rate of cells induced by DOX for 16 h in K562/ADR cells was severely prohibited when compared to K562 cells. Though matrine alone had no significant effect on neither K562 cells nor K562/ADR cells, it could dramatically enhance both the early apoptotic rate (Annexin V^+^, DRAQ 7^−^) and the late apoptotic rate (Annexin V^+^, DRAQ 7^+^) of cells in the combination of DOX.

### 3.4. Improvement of Intracellular Accumulation of Rhodamine 123 by Matrine

To examine whether matrine could reverse resistance of DOX and PTX in K562/ADR cells through inhibiting the function of ABCB1, we measured the intracellular levels of rhodamine 123, an ABCB1 substrate, in the presence or absence of matrine. As shown in Figures 1(e)–1(h), the accumulation of rhodamine 123 in K562/ADR cells was much lower than that in K562 cells. Furthermore, matrine improved the intracellular levels of rhodamine 123 in both cell lines. However, improvement in K562/ADR cells (increased by 133.6%) was much more significant than that in K562 cells (increased by 9.6%).

### 3.5. Suppressed Expression of ABCB1, ABCC1, and ABCG2 in Matrine-Treated Cells

**Figure 2:**
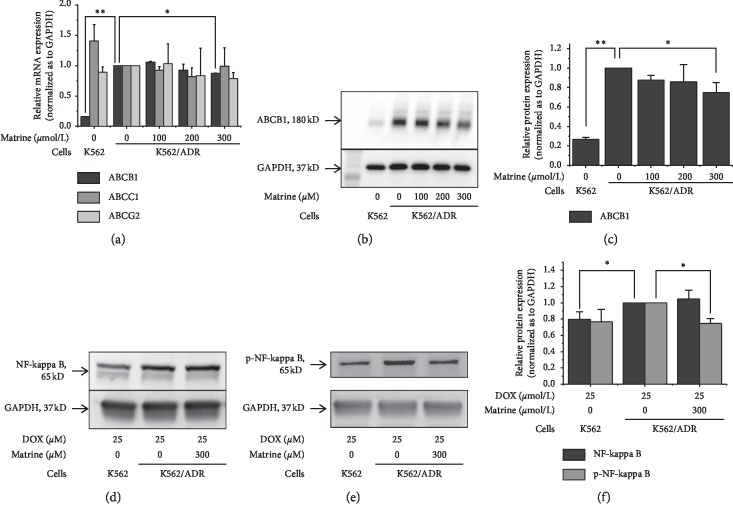
Effects of matrine on drug exporters and activation of NF-kappa B. (a) ABCB1, ABCC1, and ABCG2 mRNA expressions measured by quantitative real-time PCR analysis, 2-△△Ct assay. (b) and (c) ABCB1 protein expressions examined by western blotting. (d), (e), and (f) NF-kappa B and p-NF-kappa B protein expressions examined by western blotting. All the data were expressed as mean and SD of three independent experiments ((a), (b), and (f); ^*∗*^*P* < 0.05, ^*∗∗*^*P* < 0.01; ANOVA).

Messenger RNA levels of ABCB1, ABCC1, and ABCG2 are shown in Figure 2(a). Expression of ABCB1 mRNA in K562/ADR cells was 6.3-fold higher than that in K562 cells, while increased expression was not found in ABCC1 and ABCG2. The ABCB1 mRNA level in K562/ADR cells was decreased to 87.5% by exposure to 300 *μ*mol/L matrine.

Effects of matrine on the protein level of ABCB1 are shown in Figures 2(b) and 2(c). Protein expression of ABCB1 in K562/ADR cells was 4.85-fold high as that in K562 cells, which was decreased by 25.2% under the treatment of 300 *μ*mol/L matrine.

### 3.6. Inhibited Activation of NF-Kappa B by Matrine

The protein level of NF-kappa B in the K562/ADR cells is shown in Figures 2(d)–2(f). Under the treatment of DOX for 48 h, NF-kappa B expression was 25% higher and phosphorylated NF-kappa B was 30% (though not statistically significant) higher in K562/ADR cells in comparison with K562 cells. In the combination of 300 *μ*mol/L matrine, phosphorylated NF-kappa B was decreased to 75% in the resistant cells, which recovered to the level in the parental cells.

### 3.7. Effects of Matrine on the Apoptotic Proteins

**Figure 3:**
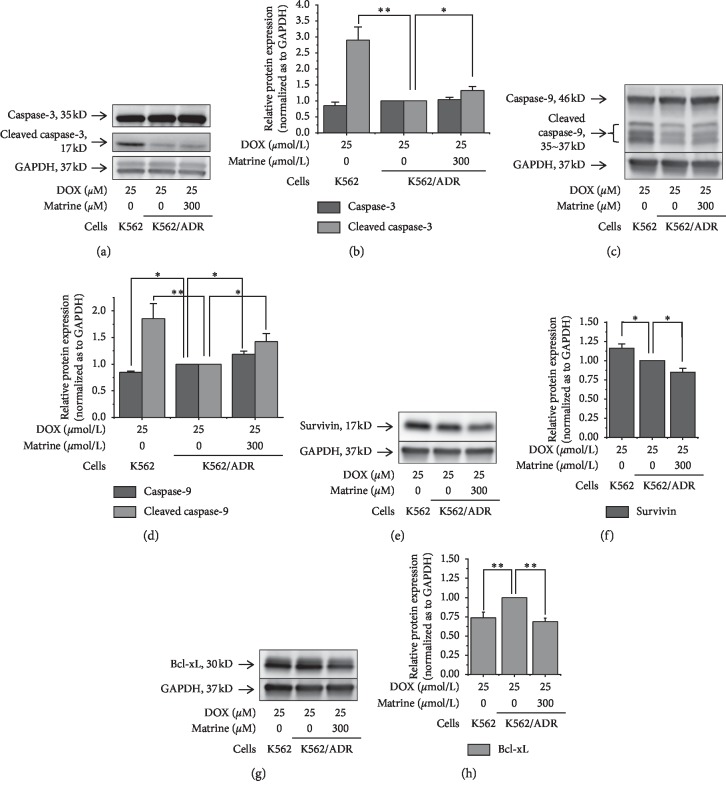
Effects of matrine on the intrinsic apoptosis-relating proteins. (a) and (b) Caspase-3, (c) and (d) caspase-9, (e) and (f) survivin, and (g) and (h) Bcl-xL. Protein expressions were examined by western blotting. All the data were expressed as mean and SD of three independent experiments ((b) (d), (f) and (h); ^*∗*^*P* < 0.05, ^*∗∗*^*P* < 0.01; ANOVA).

As an executor of apoptosis, protein expression of cleaved caspase-3 in K562/ADR cells was approximately one-third of that in K562 cells. With the treatment of 300 *μ*mol/L matrine, the expression in the resistant cells was improved by 32% (Figures 3(a) and 3(b)). As for an intrinsic apoptotic factor caspase-9, in K562 cells, the protein expression level of caspase-9 was 15% lower and that of cleaved caspase-9 was 85% higher than that in K562/ADR cells. In the combination of 300 *μ*mol/L matrine, the expression of caspase-9 and cleaved caspase-9 in resistant cells was improved by 19% and 42%, respectively (Figures 3(c) and 3(d)). Survivin, which can inhibit the caspase-3 and caspase-9 activation, was significantly suppressed by matrine in resistant cells, although the parental cells had a higher expression level than the resistant cells (Figures 3(e) and 3(f)). Expression of Bcl-xL, a suppressor of caspase-9 activation, was lower in K562 cells than in K562/ADR cells. Matrine downregulated the expression of Bcl-xL into 69% in the resistant cells, which was comparable to that of the parental cells (Figures 3(g) and 3(h)).

## 4. Discussion

Multidrug resistance is the main obstacle in chemotherapies nowadays. Many relevant studies have endeavored to search for molecular targets to solve this conundrum [8, 25]. Several chemomedicines like verapamil were found to be positive side effects that suppress drug export and recover apoptosis [26]. However, it is not used practically at the clinical level. It is possible that natural products may effectively suppress multidrug resistance in various pathways. One of the advantages of the natural product is comparatively safe to the human body, those that are effectively suppressing multidrug resistance have the potential to be further studied and evaluated [11, 19, 27]. No doubt, the safety of a medicine is an essential premise for its effectiveness. Rather than several studies using IC_20_ as the maximum concentration of multidrug resistance reversing agents [19, 28], we employed a maximum concentration even lower than IC_5_. In this study, we first assessed the properties of matrine that prevent drug efflux and facilitate apoptosis and elucidated their relationships.

Firstly, our purpose in this study is to elucidate the mechanism of ubiquitous antidrug resistance in cancer cells and how matrine suppressed it. K562 and K562/ADR cells were chosen as the model because they have been frequently used in the many research studies to elucidate possible mechanisms for multidrug resistance, especially ABC transporters [19, 20]. As anticancer drugs, we utilized DOX and PTX, which are well known to be ABCB1 substrates and induce multidrug resistance [19]. In the experiment on the reversal effect of matrine, the nontoxic concentration of matrine increased the sensitivity to DOX and PTX in resistant K562/ADR cells with the reversal fold values of 2.88 and 3.12, respectively (Table 1). These reversal effects were comparable to the other natural products reported in the previous research studies [19, 20].

ABCB1 has attracted much concern for its drug efflux properties [9, 29]. Rhodamine 123 is a well-known specific ABCB1 substrate. It has been utilized as an indicator of ABCB1 function in many relevant research studies [11, 12, 30]. Matrine in the nontoxic concentration improved intracellular rhodamine 123 levels, especially in K562/ADR cells (Figures 1(e)–1(h)). This can be well explained by the decreased expression of ABCB1 mRNA and protein by matrine (Figure 2(a)). However, we were facing two questions: first, is ABCB1 the only responsible drug-exporting factor? Second, is there any upstream factors that are regulating ABCB1 expression?

Except for ABCB1, ABCC1 and ABCG2 have been reported as the responsible membrane exporters for anticancer drug resistance. In Figure 2(a), unlike ABCB1, no significant elevation of ABCC1 and ABCG2 was found in our resistant cells with or without matrine treatment. These results suggest that suppression of ABCB1 expression by matrine is one of the mechanisms for the increased sensitivity to anticancer drugs. Though the ABCB1 level in K562/ADR cells was significantly higher than that in K562 cells, indicating high ABCB1 expression is a crucial factor for the drug resistance in this study, we can thus prove the correlation between ABCB1 and resistance of K562/ADR cells with ABCB1 knockdown cells in future works.

For tracing upstream modulators [31, 32] of ABCB1, mRNA levels of PTEN, p53, Akt, and MDM2 were also examined, but we could not find any evidence that they were responsible for ABCB1 downregulation in K562/ADR cells treated with matrine ([Supplementary-material supplementary-material-1]). On the other hand, we found overexpressed NF-kappa B and its phosphorylated form, which are believed to be promotors of ABCB1 [13, 33], in K562/ADR cells (Figures 2(d)–2(f)). Chen et al. reported that PDTC, a specific inhibitor of NF-kappa B, could downregulate the expression of ABCB1 in Caco-2 vbl cells and revealed the upstream modulating status of NF-kappa B on ABCB1 [33]. Our results showed that matrine suppressed phosphorylation of NF-kappa B in K562/ADR cells, implying that it could be responsible for the suppressed ABCB1 level. However, we did not show enough evidence to explain whether matrine downregulated ABCB1 through inhibiting NF-kappa B or inhibited ABCB1 directly in this research. Knockdown factors or inhibitors of NF-kappa B can provide more direct and convincible evidence to reveal its relation with ABCB1. Because NF-kappa B is also known to be an upstream inhibitory factor for the intrinsic apoptosis pathway [17, 18], it was necessary to test the influence of matrine on apoptosis-relating proteins.

Matrine in the nontoxic concentration enhanced apoptosis induced by DOX in K562 and K562/ADR cells though it was not effective alone (Figures 1(c) and 1(d)). To elucidate the mechanism, we examined several related proteins in the intrinsic and extrinsic apoptotic pathways. In the intrinsic apoptotic pathway, inhibited activation of caspase-9, as well as downstream executor caspases-3, can also usually lead to cancer treatment failures. Caspase-9 can be activated by proapoptosis protein Bax and antiapoptosis protein Bcl-xL. Survivin can prohibit the process of caspase-3 activation [15, 34]. According to our findings (Figure 3), activations of caspase-3 and caspase-9 were inhibited and Bcl-xL were activated in our resistant cells. Matrine inhibited Bcl-xL and survivin expression and leads to the reactivation of caspase-3 and caspase-9, partly recovered the intrinsic apoptosis. Although Bax expression did not change by matrine ([Supplementary-material supplementary-material-1]), this is consistent with upregulated caspase-9. In total, these changes in expression levels of apoptosis-relating proteins in the intrinsic pathway are considered to be another mechanism for the increased sensitivity to anticancer drugs by matrine.

The extrinsic apoptotic pathway is triggered by the interaction of exposed death receptors located on the cell surface. The activation of initiator caspase-8 can further activate the downstream executor like caspase-3 and sabotage critical substrates for cell viability, urging cell death [15]. Nevertheless, we could not find the difference of the caspase-8 level, which is vital in the extrinsic apoptotic pathway ([Supplementary-material supplementary-material-1]). These results indicate that matrine does not affect the extrinsic apoptosis pathway but affects the intrinsic pathway.

NF-kappa B is an upstream factor that activates Bcl-xL and thus inhibits caspase-9, which plays a crucial role in handicapping the intrinsic apoptotic pathway [17, 18]. Because matrine suppressed the activation of NF-kappa B (Figures 2(d)–2(f)), we assumed that suppressed antiapoptotic proteins and recovered activities of proapoptosis protein were involved with NF-kappa B inactivation and thus recover the intrinsic apoptotic pathway exclusively. Our hypothesis is supported by several relevant research studies. Shao et al. implied suppression of inhibitor of kappa B kinase *β* by matrine might lead to suppressed NF-kappa B activation [35]. Zhou et al. found matrine induced cell death in HepG2 cells through facilitating the intrinsic apoptotic pathway [3]. Luo et al. reported matrine suppressed survivin expression in NCI-H520/TAX25 cells, also implying it could facilitate the intrinsic apoptotic pathway [6]. Zhou et al. indicated matrine could facilitate the intrinsic apoptotic pathway by downregulating antiapoptotic factor Bcl-2 [7].

Although the effect of matrine on either ABCB1 or apoptosis-related proteins is small (about 20–30% compared to control), the collective effect of them can lead to a noticeable weakened multidrug resistance, which can be observed through IC_50_s.

Natural products derived from herbal medicines are broadly synthesized into compounds with anticancer or multidrug resistance reversing activities [36]. It is important that detailed mechanisms for anticancer and multidrug resistance reversing properties as well as safety in these compounds are investigated.

## 5. Conclusion

In conclusion, our study demonstrated that matrine in the nontoxic concentration resensitized multidrug-resistant K562/ADR cells in two ways: reactivating apoptosis and inhibiting drug efflux. Matrine can downregulate phosphorylation of NF-kappa B to recover proapoptotic factor and suppress antiapoptotic factors, leading to facilitated intrinsic apoptosis. In addition, matrine can downregulate ABCB1 expression to induce diminished drug efflux, which may be also related to the suppressed NF-kappa B.

## Figures and Tables

**Table 1:** Determination of IC_50_s of DOX and PTX in the combination of different concentrations of matrine. Cell viabilities were examined by WST-8 assay. IC_50_s were calculated. Reversal fold = IC_50_s for cells treated with matrine/IC_50_s for cells treated without matrine. Each value of IC_50_ represented as mean and SD of three independent experiments.

Cells	Concentration of matrine (*μ*mol/L)	IC_50_ of DOX (*μ*mol/L)	Reversal fold
*DOX*			
K562	0	2.75 ± 0.9	
	200	2.38 ± 1.11	1.16
	300	2.77 ± 1.66	0.99
K562/ADR	0	73.63 ± 2.37	
	200	31.99 ± 4.31	2.3
	300	25.53 ± 1.28	2.88

*PTX*		IC_50 _of PTX (nmol/L)	
K562	0	793 ± 149	
	200	692 ± 77	1.15
	300	629 ± 37	1.26
K562/ADR	0	7990 ± 561	
	200	2789 ± 284	2.86
	300	2563 ± 196	3.12

## Data Availability

The data used to support the findings of this study are available from the first author or corresponding author upon request.

## Abbreviations

ABC: Adenosine triphosphate-binding cassette
Bcl-2: B-cell lymphoma 2
Bax: Bcl-2-associated X protein
DOX: Doxorubicin
FBS: Fetal bovine serum
MDM2: Mouse double minute 2 homolog
PTX: Paclitaxel
PTEN: Phosphatase and tensin homolog
IC_50_: The concentrations required to inhibit growth by 50%.

## Supplementary Materials

Supplementary Materials Table S1: primers for quantitative real-time PCR. Figure S1: matrine did not influence mRNA expressions of PTEN, p53, Akt, or MDM2 in K562/ADR cells. Figure S2: matrine did not suppress Bax expression in K562/ADR cells. Figure S3: matrine did not induce activation of caspase-8.
