# Supplementary material for: Molecular Mechanism of Matrine from Sophora alopecuroides in the Reversing Effect of Multi-Anticancer Drug Resistance in K562/ADR Cells
Source: Biomed Res Int. 2019 Nov 22;2019:1269532. doi: 10.1155/2019/1269532 (PMC6906886; doi:10.1155/2019/1269532)
Supplement: Supplementary Materials — Table S1: primers for quantitative real-time PCR. Figure S1: matrine did not influence mRNA expressions of PTEN, p53, Akt, or MDM2 in K562/ADR cells. Figure S2: matrine did not suppress Bax expression in K562/ADR cells. Figure S3: matrine did not induce activation of caspase-8. [file 1269532.f1.zip › 1269532.f1/Fig. S2.pdf]

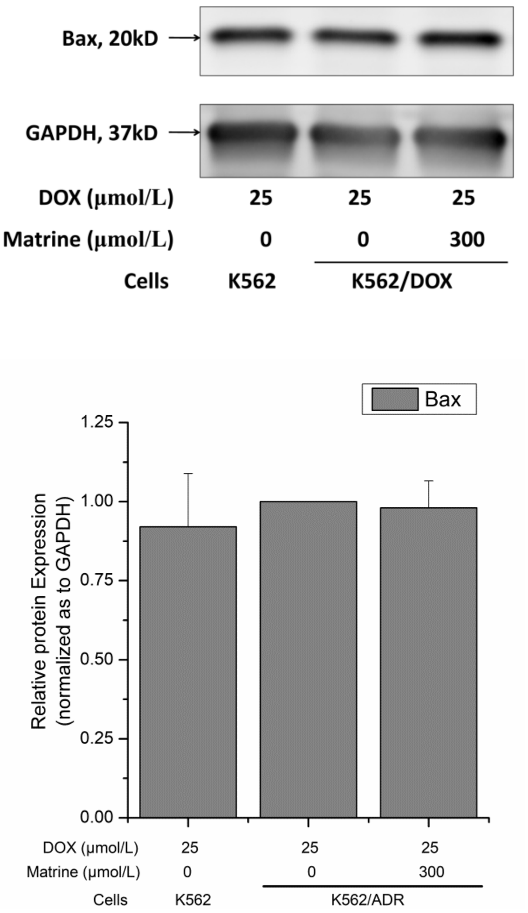

Fig. S2 Matrine did not suppress Bax expression in K562/ADR cells. K562 cells and K562/ADR cells with 25μmol/L doxorubicin (DOX) and 0 or 300 μmol/L matrine were cultured for 48 h. Total protein of Bax was extracted for western blotting analysis. The density of bands was measured and employed as quantification of protein. All the data were expressed as mean ± SD of three independent experiments.
