# Supplementary material for: Molecular Mechanism of Matrine from Sophora alopecuroides in the Reversing Effect of Multi-Anticancer Drug Resistance in K562/ADR Cells
Source: Biomed Res Int. 2019 Nov 22;2019:1269532. doi: 10.1155/2019/1269532 (PMC6906886; doi:10.1155/2019/1269532)
Supplement: Supplementary Materials — Table S1: primers for quantitative real-time PCR. Figure S1: matrine did not influence mRNA expressions of PTEN, p53, Akt, or MDM2 in K562/ADR cells. Figure S2: matrine did not suppress Bax expression in K562/ADR cells. Figure S3: matrine did not induce activation of caspase-8. [file 1269532.f1.zip › 1269532.f1/Fig. S3.pdf]

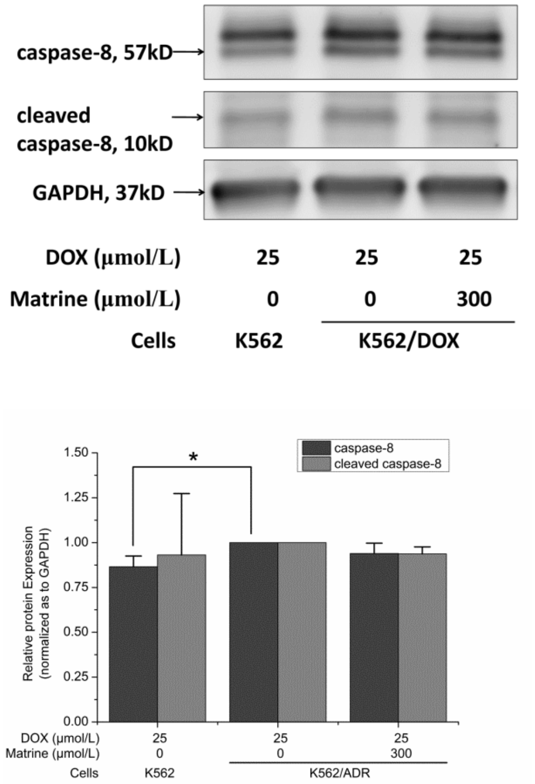

Fig. S3 Matrine did not induce activation of caspase-8. K562 cells and K562/ADR cells with 25μmol/L doxorubicin (DOX) and 0 or 300 μmol/L matrine were cultured for 48 h. Total protein of caspase-8 and cleaved caspase-8 was extracted for western blotting analysis. The density of bands was measured and employed as quantification of protein. All the data were expressed as mean ± SD of three independent experiments (*P < 0.05).
