# Supplementary material for: Molecular Mechanism of Matrine from Sophora alopecuroides in the Reversing Effect of Multi-Anticancer Drug Resistance in K562/ADR Cells
Source: Biomed Res Int. 2019 Nov 22;2019:1269532. doi: 10.1155/2019/1269532 (PMC6906886; doi:10.1155/2019/1269532)
Supplement: Supplementary Materials — Table S1: primers for quantitative real-time PCR. Figure S1: matrine did not influence mRNA expressions of PTEN, p53, Akt, or MDM2 in K562/ADR cells. Figure S2: matrine did not suppress Bax expression in K562/ADR cells. Figure S3: matrine did not induce activation of caspase-8. [file 1269532.f1.zip › 1269532.f1/Table S1.pdf]

Table S1 Primers for quantitative real-time PCR

Gene	Accession NO. in Genbank	Forward/Reverse	Sequence (5' to 3')	Tm (℃)	Annealing Temperature (℃)
GAPDH	NM_002046.7	Forward 249-268	GCACCGTCAAGGCTGAGAAC	61.57	60
		Reverse 386-368	TGGTGAAGACGCCAGTGGA	61.14	60
ABCB1	NM_001348946.1	Forward 3592-3611	AGGCCAACATACATGCCTTC	58.23	60
		Reverse 3676-3657	CCACCAGAGAGCTGAGTTCC	59.75	60
ABCC1	NM_004996.4	Forward 3427-3446	CCTGTTCAACGTCATTGGTG	57.31	60
		Reverse 3541-3522	AGCCACGTAGAACCTCTGGA	60.25	60
ABCG2	NM_004827.3	Forward 710-729	TTCGGCTTGCAACAACTATG	57.01	60
		Reverse 837-818	TCCAGACACACCACGGATAA	58.37	60
PTEN	NM_001304718.2	Forward 1877-1901	GAGCGTGCAGATAATGACAAGGAAT	61.71	60
		Reverse 2028-2004	GGATTTGACGGCTCCTCTACTGTTT	62.82	60
p53	NM_014477.3	Forward 853-873	ACAAGGTTGATGTGACCTGGA	59.23	60
		Reverse 957-937	TGTAGACTCGTGAATTTCGCC	58.4	60
Akt	NM_001626.6	Forward 247-266	ACGATGAATGAGGTGTCTGT	56.57	60
		Reverse 425-406	TCTGCTACGGTGAAGTTGTT	57.1	60
MDM2	NM_002392.5	Forward 1625-1648	CCATTGAACCTTGTGTGATTTGTC	59.02	60
		Reverse 1739-1715	TTCCTTTTCTTTAGCTTCTTTGCAC	58.73	60

Primers we used in quantitative real-time PCR was synthesized by Eurofins Genomics (Tokyo, Japan). The data was from Genbank and BLAST.
